# Animal models of hemorrhage, parameters, and development of hemostatic methods

**DOI:** 10.1186/s42826-025-00239-5

**Published:** 2025-02-03

**Authors:** Gholamhossien Darya, Hamid Mohammadi, Zeinab Dehghan, Alireza Nakhaei, Amin Derakhshanfar

**Affiliations:** 1 Department of Comparative Biomedical Sciences, School of Advanced Medical Sciences and Technologies, Shiraz University of Medical Sciences, Shiraz, Iran; 2 Department of Pediatric, School of Medicine, Shiraz University of Medical Sciences, Shiraz, Iran; 3 Autoimmune Diseases Research Center, Shiraz University of Medical Sciences, Shiraz, Iran

**Keywords:** Hemorrhage, Bleeding, Animal modeling, Hemostat

## Abstract

Hemorrhage is a prevalent side effect of various injuries and can be life-threatening in certain instances. It is categorized into compressible and non-compressible types, each necessitating distinct modeling, laboratory assessments, and hemostatic approaches. This study utilized the keywords Hemorrhage, Bleeding, Animal Modeling, and Hemostat in reputable databases. The findings indicate that femoral artery hemorrhage and hepatic parenchymal hemorrhage are the predominant modeling techniques for compressible and non-compressible bleeding, respectively. Furthermore, it is noted that animal models of compressible hemorrhages are primarily situated in superficial body areas to investigate dressing or additive hemostats, while non-compressible hemorrhage models, typically located in visceral organs, are employed to examine adhesive or surgical instrument-based hemostats.

## Background

Blood is a specialized connective tissue that facilitates the transmission of various elements and supports organ functions to maintain homeostasis within the vascular network. Hemorrhage refers to any damage to the vascular network that results in bleeding [1, 2]. This process may significantly influence physiological homeostasis [3]. The clinical consequences of hemorrhage vary based on the duration of bleeding and physiological storage capacity. Abnormal bleeding leads to cardiovascular failure, impaired tissue oxygenation, and organ dysfunction. Rapid progression of this lesion may result in cerebral and cardiac ischemia, potentially leading to coma, cardiac arrest, and ultimately death [4].

Hemorrhage ranks as the primary and secondary cause of mortality in battlefield and accident scenarios, respectively [5]. Hemorrhage constitutes approximately 80% of treatable war injuries. Additional statistics revealed that 15% of traumas in the general population are associated with severe bleeding, and of these shock injuries, 45% resulted in mortality [6]. Trauma with hemorrhagic complications is classified into two categories: compressible and non-compressible. Non-compressive trauma and uncontrolled cutaneous bleeding represent two critical forms of lesions that pose significant threats to life. Applying pressure to the injured area is regarded as the primary treatment for cutaneous bleeding. Moreover, it is established that when pressure surpasses blood pressure, it aids in halting bleeding and significantly alleviates organ dysfunction and ischemia [2]. Significant non-compressible injuries encompass pulmonary injury, fourth-degree solid visceral injury, major axial vessel injury, and pelvic ring injuries associated with fractures [4]. In addition to surgical interventions for hemorrhage control, there is a necessity for non-surgical treatment options, particularly in locations where surgical procedures are not feasible, such as combat zones. Severe hemorrhage during invasive surgical procedures represents an additional condition. The acceleration of wound closure or healing is of significant importance in the field of medicine. Conventional sutures present disadvantages such as being time-consuming, failing to provide in-situ hemostasis, and causing lateral soft-tissue damage [7].

Modeling hemorrhage can be defined as the creation of a wound resulting from the rupture of blood vessels within the organ parenchyma, arteries, or major veins, leading to blood loss. These models must exhibit several characteristics, including reproducibility, the ability to consistently assess blood loss during experimental tests, monitoring of blood dynamics and physiological responses during hemorrhage, and analysis of the interactions between hemostatic agents and circulating blood cells and tissue. Nonetheless, creating a stable animal model with the aforementioned characteristics remains a significant obstacle to advancing experimental research on bleeding [6, 8].

The femoral hemorrhage model is the predominant model utilized for developing treatment strategies for trauma and superficial lesions, specifically referred to as compressible hemorrhagic lesions. The selection is justified by easy accessibility, appropriate pressure, and the absence of significant hazards, such as vessel ruptures in the neck region, for the animals under study. Rabbits and agricultural livestock Are ideal laboratory animals utilized in this modeling [9, 10]. The subsequent option for modeling cutaneous hemorrhage involves a tail incision in Syrian mice and rats. Although the implementation process is straightforward, the applicability of results to humans is limited [11, 12]. Nonetheless, the underlying rationale in the development of these animal models is to mitigate mortality associated with blood volume and cardiac output reduction during the initial minutes of hemorrhage. This is achieved by either minimizing substantial blood volume loss from the vessels or by examining hemodynamic responses and physiological reactions to considerable blood volume depletion [13, 14].

Achieving control of bleeding within the shortest amount of time is an undeniable proficiency for rescuers and therapists during and after emergency. Consequently, enhanced comprehension of hemostatic drugs and technical proficiency are essential for managing various animal models of hemorrhage. This research paper aims to examine prevalent bleeding models, laboratory tests, methodologies, and materials commonly utilized in hemorrhage studies.

## Main text

Articles on hemorrhage modeling and treatment were searched in Google Scholar, Web of Science, Scopus, and PubMed databases. The subject terms used included bleeding or hemorrhage or hemostat and animal model or rodent or domestic animal or large animal or specific name of conventional laboratory animal as the retrieved articles were further analyzed. In the following, invalid articles, coagulopathy articles and repetitive modeling were removed.

### Types of experimental hemorrhages in animal models

#### Cutaneous hemorrhage

##### Vena femoralis/Arteria hemorrhage modeling in rat

The laboratory rat (*Rattus norvegicus*) is known as the most conventional experimental animal. Cardiovascular diseases represent a prominent area of research utilizing this animal in biomedical studies [15]. An examination of various research articles indicated that the appropriate weight of a rat for the bleeding model should fall within the range of 200 to 450 g. 2–5% isoflurane, 2–3% halothane, Pentobarbital, and ketamine are currently used for anesthesia induction. Additionally, during the surgical procedure, it is essential to closely monitor the end-tidal concentration of carbon dioxide and halothane using capnography, while ensuring that the temperature is maintained at 37 °C [16]. The ideal temperature can be obtained using a straightforward lamp in the rat. Hair removal by shaving in conjunction with applying disinfection procedures is a prelude to surgery. Surgery commences with a transverse incision on the femoral canal, followed by dissection measuring 20–25 mm to separate arteries, veins, and nerves from one another. Furthermore, the identical procedure is utilized for the anterior femoral canal to a cannula. Furthermore, it was recommended to collect several key parameters, including MAP (Mean Arterial Pressure) and other hemodynamic variables [16].

##### Modeling of femoral artery hemorrhage in rabbit

The White New Zealand Rabbit (*Oryctolagus cuniculus*) is the most commonly used in-vivo model for surgical, orthopedic, and cardiovascular research [17, 18]. The weight of the animals used in this experimental study ranged from 2 to 4 kg. The establishment of the same model in rabbits necessitates the use of injectable anesthetic, specifically sodium pentobarbital at a dosage of 40–45 mg/kg, or a combination of ketamine at 35 mg/kg with xylazine at 5 mg/kg. A xylazine injection of 0.2 ml/kg is recommended for rabbits. The surgical procedure is identical for both large and laboratory animals; however, the surgical incision measures approximately 5 cm in length along the inguinal region [19–21].

**Fig. 1:**
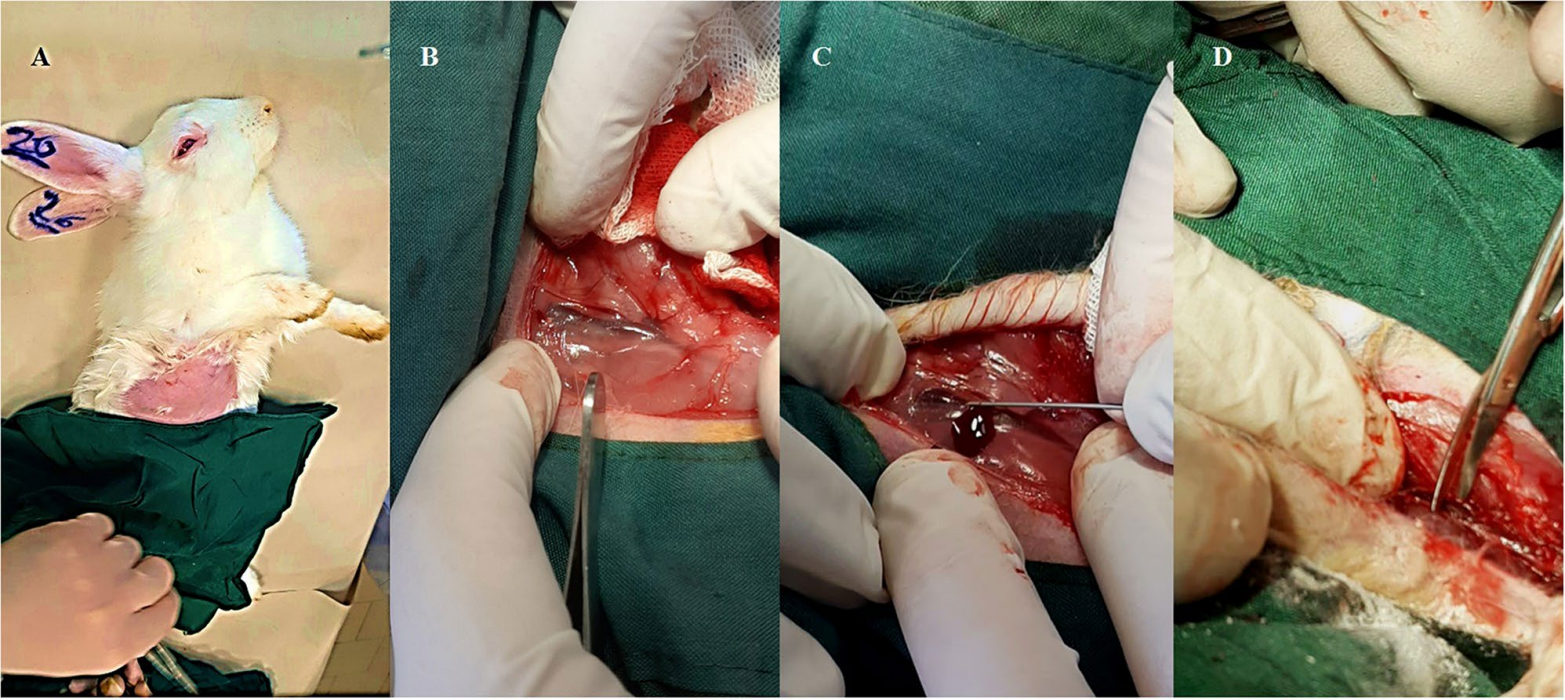
Shaving, scrubbing and draping on the inguinal area of the anesthetized rabbit (**A**); skin incision and fascial dissection to expose the femoral vein (**B**); sub-lethal bleeding formation, using needle stick (**C**), lethal bleeding as a result of femoral artery and vein section by incisor (**D**)

To evaluate various hemostatic agents, the authors established models of sublethal and lethal femoral bleeding. The femoral vein and artery were exposed following the preparation and draping of the inguinal region. In the initial assessment, a 3 mm incision in the femoral vein using a 20-gauge needle served as the sublethal bleeding model, while a complete transection of the femoral artery was employed for the lethal bleeding model. For the hemostatic evaluation, hemostatic powders were applied, and manual pressure was exerted on the site. The cessation of bleeding was monitored at intervals of 30–60 s for the sublethal and lethal models, respectively (Fig. 1) [22].

##### Modeling of femoral artery hemorrhage in sheep

The findings from large animal experiments are more reliable, with sheep (*Ovis aries*) being a preferred choice for testing surgical facilities, particularly in the area of cardiovascular diseases [23]. The animals involved in this research study had weights ranging from 7 to 9 kg, and they were provided 15 days to acclimate to the new environment. Anesthesia induction for the surgical procedure was accomplished using a 5 mg/kg ketamine injection, followed by maintenance with inhalation of 2–5% isoflurane [13].

Arterial blood pressure is continuously recorded, and blood sampling is conducted through the placement of arterial and venous lines in both the left and right femoral arteries. Temperature-resistant vascular catheters, exemplified by the Swan-Ganz thermodilution catheter (7 F), are inserted into the pulmonary artery to assess arterial blood pressure, central venous pressure, and intermittent cardiac output. Furthermore, to mitigate the risk of blood clots, a transducer measuring 4 × 4 is inserted into the tubes and catheters to facilitate the continuous infusion of heparin detergent at a rate of 3 ml/h per tube into the vein. During the surgical procedure, body temperature, total blood cell count, and any discomfort or pain will be monitored closely. Buprenorphine is recommended as a sedative agent, and due to fluid loss during hemorrhage, a Ringer’s lactate solution at a rate of 2 ml/kg/h is advised [24].

In this hemorrhage modeling, blood flow to the surgical site is absent, and experimental bleeding is simulated using a tubing rotary pump at two rates: fast (1.25 ml/kg/min) and slow (0.25 ml/kg/min) [13].

##### Modeling of femoral artery hemorrhage in pig

The use of large animal models in biomedical studies is likely to enhance the generalizability of findings from animal studies to human populations. Pigs (Sus domesticus) exhibit hemato-physiological and cardiovascular similarities to humans among large animals, excluding non-human primates. Notably, the most comparable contusion has been documented in this species, particularly in young males [25, 26]. For this procedure, pigs should weigh between 25 and 35 kg and undergo a fasting period of 8 h prior to the experiment. The surgical procedure must be conducted under general anesthesia. This experimental study commences anesthesia induction through the intramuscular administration of 30 mg/kg ketamine. Additionally, inhalation of 3 to 4% isoflurane was sustained via the tracheal tube or with the aid of a respiratory mask [14]. Vascular catheters sized 18 to 20, along with a 7–9 F introducer, are utilized for the monitoring of vital signs by being inserted into the carotid artery and the right internal jugular vein, respectively. Furthermore, pulse oximetry was utilized to gather blood oxygen measurements from the tongue, tail, and ear. During bleeding, body temperature decreased and was measured rectally, remaining between 37 and 39 °C with the use of a Bair Hugger heater [14]. In this large animal model, the groin area dimensions may be increased by up to 12 cm. Additionally, administering 1 to 2% lidocaine injection into the vein and blood vessels proximal to the surgical site is performed to minimize bleeding. This approach ensures that blood flow remains consistent, allowing for the maximum volume of blood to be collected. Prior to the vessel piercing, position a bulldog clamp on top and create a 4 mm aperture. The zero-bleeding time will align with the removal of the clamp. Treatment may commence 45 min post-hemorrhage. Additionally, blood leakage at the injury site and vital signs are monitored every 15 min for the subsequent three hours. At the conclusion of the third hour, the surviving animals were euthanized through the injection of eutanazol solution [27].

##### Tail hemorrhage modeling in rats

The tail-bleeding model is restricted to rats and Syrian mice. This method is analogous to other techniques, and the surgical procedure will be performed under general anesthesia. The distal one-third of the tail, specifically 4 cm in rats and 1 to 2 cm in Syrian mice, is an optimal location for scalpel incision. The suitable weight for rats aligns with that observed in other models, typically ranging from 25 to 35 g for Syrian mice [28, 29]. Tail bleeding serves as a supplementary assay in conjunction with other hemorrhage models to evaluate coagulation and clotting parameters prior to or following the primary model [30].

##### Hemorrhage modeling in ear

Hemorrhage from the skin or ears of laboratory animals is frequently employed to investigate specific forms of bleeding. General anesthesia is also necessary. Removal of animal hair is crucial for accurate access to the ear arteries in rabbits. Notably, 100% of hand arterial blood pressure can be obtained through whole layer puncture generation in the ear [20, 21].

Furthermore, the authors are designating the opposite ear as a control group. The extensive development of blood vessels in this animal enables the generation of bleeding through arterial and venous blood or the selective rupture of a vessel [22].

Rats and Syrian mice are primarily utilized for controlled hemorrhage studies. Selecting a bleeding site near the middle longitudinal axis of the ear is recommended to improve the test’s repeatability [20].

##### Skin hemorrhage modeling

full thickness excisional injuries to skin without damage to the main artery do not trigger significant and uncontrollable bleeding. skin surgical incision was carried out to investigate the interaction between understudy hemostatic agents with a living organism and its antimicrobial capabilities [7, 31].

### Modeling of visceral-surgical hemorrhage (non-compressible)

This section emphasizes the modeling of inaccessible and non-compressible organ hemorrhage. In this scenario, controlling bleeding and addressing organ failure presents significant challenges. Deep hemorrhage from the trunk exemplifies uncontrolled hemorrhage, necessitating surgical intervention. Given that hemorrhage is a time-sensitive injury, achieving success prior to irreversible ischemia has been a longstanding objective for clinicians [32].

#### Modeling of liver hemorrhage in Syrian mice

The animals utilized in this experimental research must weigh between 25 and 35 g and be at least 5 weeks old. A longitudinal incision along the midline is advised for this hemorrhage model. Disinfection of the surgical site and the administration of general anesthesia are prerequisites for surgical intervention. After the excision of the skin and abdominal wall, the liver becomes the first organ observable. To assess the volume of blood lost during a surgical procedure, it is prudent to excise a small portion of an accessible lobe from the ventricular site, ensuring that adjacent organs remain undamaged during the operation. Ophthalmic scissors can facilitate the removal of 3.5 mm tissue through the entire length of parenchyma tissue [33].

#### Modeling of liver hemorrhage in rat

**Fig. 2:**
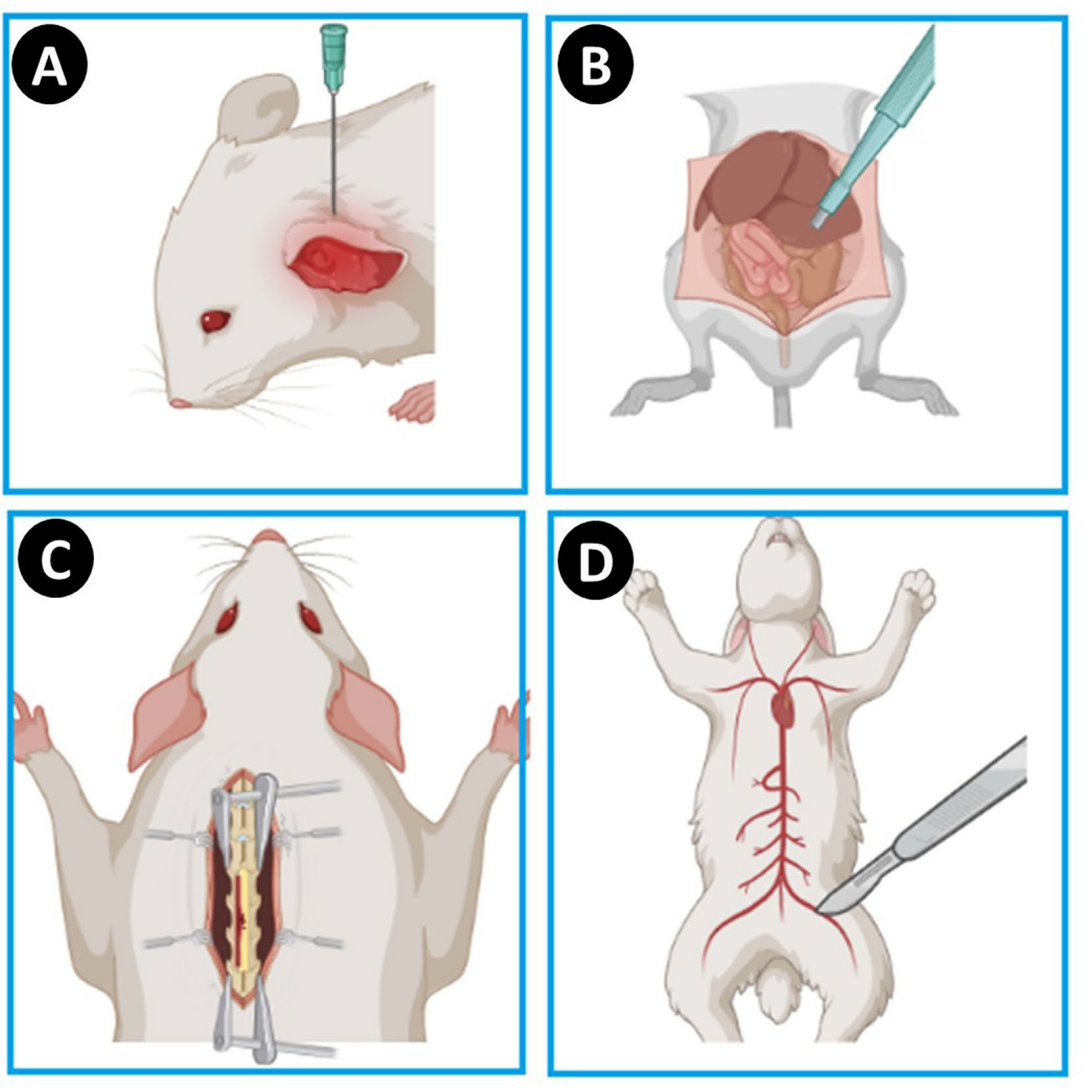
Ear hemorrhage modeling (**A**); Visceral hemorrhage modeling owing to puncture the left lateral lobe through biopsy punch (**B**); modeling the injured site through a microscopic laminectomy of dura mater and vertebrates with the help of a high-speed drill, immediately after subcutaneous and muscle resection (**C**); sub lethal to lethal hemorrhage modeling via major vessels (femoral vessels) cutting off

Animals participating in this experimental study must be 5 to 7 weeks old. The animal should be positioned on the surgical operating table, with its tail fixed against the veterinary surgeon’s face. Additionally, it was shown that the left lateral lobe of the liver is the first identifiable region of the liver during laparoscopic surgery of the abdominal wall. A biopsy punch measuring 12 mm in diameter and 2 mm in depth may be utilized to effectively model various groups. The laceration model represents an experimental approach to liver hemorrhage that closely resembles the actual clinical condition. Consequently, the margin of the left liver lobe measuring 3 × 1.5 cm may be incised, and hemostatic studies can be conducted (Fig. 2) [28].

#### Modeling of hepatic hemorrhage in pig

Pigs possess several characteristics, including (1) they are physiologically and anatomically very similar to humans, and (2) the liver’s large surface area facilitates multiple injuries and repeatability of the test, which made it an appropriate option for modeling hepatic hemorrhage. In this modeling system, the ideal age and weight ranges are 16 to 20 months and 50 to 70 kg, respectively. Furthermore, usual midline abdominal surgical incision led to the creation of multiple hemostatic and hemorrhage sites in different lobes, such as the left lateral, middle left, and right middle [34]. In a well-designed study that aims to remove a large amount of the liver, the removal area can be marked with electric cautery and we could through a laparoscopic stapler perform transection at the free edge of the lobes [35].

#### Modeling of hepatic hemorrhage in dog

The dog serves as the primary animal model for studying hemorrhage within large animals. Its size facilitates a manageable surgical field for the development of trauma and hemorrhage models and their corresponding management strategies [36]. The surgical technique employed mirrors that of a midline abdominal approach, with the notable distinction that the caudate lobe is more accessible. For optimal results, dogs weighing between 15 and 20 kg are recommended for creating an injured site of 3 × 4 cm with a depth of 1.5 cm. Administering antibiotics, such as ceftriaxone (500 mg intramuscularly), is a critical practice for preventing postoperative infections following the surgical intervention [37].

#### Modeling of splenic hemorrhage in the rat

The spleen is identified as the second target in cases of frequent trunk trauma, where its hemorrhage poses a significant threat to life. Moreover, it was revealed that bleeding represents the most severe adverse effect linked to both complete and incomplete splenectomy, with an associated mortality rate of 100% [38, 39]. Similar to liver modeling, the surgical approach is midline abdomen. A 3 cm incision adequately provides access to the spleen in the rat specimen. The spleen is located at the lower pole of the stomach, and a one-centimeter incision the parenchyma results in considerable bleeding [40].

#### Modeling of splenic hemorrhage in pig

The optimal weight of animals in this experiment should be about 24 kg and the surgical approach is midline abdominal. Based on Fonouni et al. (2018), surgical incisions of 2 cm in length, 2 mm in depth, and 10 cm away from the lower end of the spleen and perpendicular to its longitudinal axis are recommended. Furthermore, during surgical procedures, vital circulatory-related symptoms such as heart rate, main arterial pressure, and central venous pressure can be evaluated via catheterization superficial electrodes, in the carotid artery, and the cervical vein [35].

#### Modeling of splenic hemorrhage in dog

Animals weighing between 7 and 11 kg were selected for this modeling. Anesthesia induction involves the administration of 25 mg/kg ketamine combined with 20 µg/kg medetomidine, followed by maintenance with 1–2% isoflurane inhalation. During the surgical procedure, the animal is positioned in a dorsal recumbent posture. A midline abdominal incision measuring 15 to 20 cm is made from the midline of the sternal appendage to the pubic bone. Prior to the initiation of surgical procedures, it is essential to identify the arteries supplying the spleen. Subsequently, an asymmetrical partial splenic resection is performed, resulting in the division of the spleen into two sections. Additionally, it was demonstrated that dogs permit an increase in test repetitions by executing a similar incision on a larger segment [41].

#### Renal hemorrhage modeling in rabbit

The paired nature of the kidneys allows for the possibility of each undergoing the surgical procedure in a repeated manner across different groups. A midline incision is advised, and by carefully separating the limbs, access to the kidneys can be achieved. Additionally, it was found that hemorrhage is frequently linked to a surgical incision at the dorsal edge measuring 15 mm in length and 5 mm in depth [42, 43].

Blood entering to the renal umbilicus faces the assessment of hemorrhage volume with challenges. However, the hemostatic response of the main vessel affected by severe trauma is crucial for modeling purposes. Hemorrhages in this region will remain consistent with previous observations [44].

#### Modeling of pulmonary hemorrhage in dog

Severe bleeding from the pulmonary artery and its main branches during pulmonary lobe resection surgeries presents significant challenges and risks; thus, it is crucial to model and investigate hemostatic methods [45].

Frequently employed medicine for anesthesia induction in canines consist of pre-anesthesia using 0.2 mg/kg of Butorphanol combined with 0.06 mg/kg of Acepromazine via subcutaneous injection, induction of anesthesia with 4 mg/kg of Propofol, and maintenance of anesthesia utilizing isoflurane. During surgical sedation, a single dose of 0.2 mg/kg of meloxicam is commonly administered alongside a subcutaneous injection of ketamine, lidocaine, and fentanyl. Additionally, in the presence of thoracoscopic visualization, bupivacaine may be administered in the intercostal space to achieve blockade of the spinal nerves at the surgical site. Post-surgery and upon regaining consciousness, it is recommended to administer a subcutaneous injection of 0.01 mg/kg buprenorphine and 0.1 mg/kg meloxicam for a duration of up to six days, contingent upon the observation of clinical symptoms such as pain and restlessness [24].

Since the right thoracic space in a dog is wider and the surgeon has greater mobility, it is appropriate for modeling initiation. Upon chest area opening, the surgeon team assessing determines which of the upper or lower lobe is appropriate to be respected. To maintain the animal’s respiration during surgery, tracheal intubation with a tracheal tube (11 mm in diameter), is the preface of the operation. Next, the left trachea is endoscopy with the aim of recognizing anatomical diversity, and finally, a 5 mm trachea tube passing through a 7 mm tube is sent into the left lung. This strategy is commonly used since dog airways length is longer than humans. Furthermore, an Intubation introducer can also be used to facilitate the preceding steps. Procedures frequently performed for removing the lobe of the lung by Video-Assisted Thoracoscopic Surgery (VATS) in dogs follow the same surgical procedures in humans. Firstly, the animal received an intravenous infusion of cefazolin (22 mg/kg) to prevent infection. The animal should be placed on the surgical operative table in the form of a left lateral recumbent. Front - the axial line between the ribs 8 and 9 is the initial entry of the thoracic for camera entrance [46]. The second entrance is made in the space between the sixth ribs backward and for accessibility of the surgical area; the third entrance is created in the space between the fourth or fifth ribs. Before any hemorrhage modeling, removing the common sheath of the vein, pulmonary artery, and pulmonary capillaries of individual lobes or the lobe’s connections by monopolar cautery is suggested. Vessels frequently used for hemostatic studies as well as removed lobes should all be marked with colored vessel loops before incision. It is recommended to block the airways or blood vessels that are not involved in the study with 35 mm surgical endopathic staples because all vessels and bronchi would be incised during the lung lobe resection procedure. Additionally, the diameter of the vessels should be measured before implementing any hemostatic method through sterile calibrated paper with the aim of accurate quantification of the hemostatic method [24]. If the study continues for a long time, Blake drain is placed at the anterior-inferior entrance to drain the contents for 10 min. Furthermore, air has to be evacuated by placing a one-way Heimlich valve at the end [24].

#### Hemorrhage modeling in Anastomosis area

The anastomosis area pertains to the rupture of hollow organs, particularly the major arteries within the body. High blood pressure and its location at the axial depth of the body complicate the management of hemorrhage and hemostasis, increasing sensitivity in these processes. Suturing serves as the primary method for managing bleeding in anastomosis areas; however, emergency situations present distinct challenges. Rats are a readily available species for conducting this model. The descending portion of the aorta in the abdominal region, prior to the bifurcation known as the iliac, is considered the optimal site and vessel for this method of study [47].

**Hemorrhage modeling in bone marrow**: Recent research papers have identified rats as a viable model for bone marrow studies. The cranial bone was identified as the suitable region for this model. Skin and hair removal is unequivocally supported by standard guidelines. Trephine drills with a radius of 3 mm and a speed of 1500 rpm are essential for creating injuries with consistent depth and dimensions across all groups. The selection of this area offers several advantages: (1) the presence of dura mater beneath the skull bone, which protects the underlying soft tissue from injury; (2) minimal disruption to animal feeding or mobility during the experimental study; and (3) the ability to generate twin lesions in the parietal bone on both the left and right sides of the head [48].

#### Hemorrhage modeling in spinal vasculature

Hemorrhage is the most anticipated complication and potential risk associated with surgical procedures. Investigating hemorrhage, including minor instances during surgery, is valuable for multiple reasons. First, even minimal blood loss in the operating room can impair the surgeon’s precision; second, traditional hemostasis procedures can be inefficient and often necessitate specialized equipment; third, the adherence of blood clots to surgical instruments leads to diminished work quality, challenges in cleaning, and depreciation; fourth, the infiltration of blood cells into the central nervous system, marked by a reduction in blood cell count, correlates with heightened postoperative complications and prolonged surgical recovery [49].

Positioning the animal supine is advantageous for accessing the L4 or L5 vertebrae for incision. After the excision of skin and subcutaneous tissue, muscles are extracted using a surgical burr. Subsequently, to access the hard tissue of the jaw and to prepare the environment for hemostatic agent testing, lamina bone measuring 7 × 4 mm is excised using a surgical drill (Fig. 2) [49].

### Monitoring and maintenance

All methods of modeling hemorrhage are undeniably invasive. Tissue rupture, blood vessel perforation, and extremity amputation can induce significant stress. Blood prolactin and cortisol measurements indicate that humans in comparable conditions experienced lower stress levels than their laboratory counterparts [50, 51]. Hemorrhage-related stress is typically categorized into two components: anesthesia induction and the surgical procedure, along with its impact on the overall condition of the animal. Moreover, it was shown that the type of animal significantly affects test outcomes due to these stresses. Understanding fundamental strategies for anesthesia and recognizing each animal’s sensitivity to critical conditions, such as hemorrhage, are essential for making informed decisions and preventing experiments under uncontrolled conditions [51].

### Physiologic response to hemorrhage

Minor blood loss does not correlate with clinical manifestations. However, a 10% reduction in total blood volume (TBV) would trigger responses via baroreceptors and the adrenal gland. The primary clinical signs include: (1) elevated heart rate, (2) contraction of the arterial bed in muscles and skin, and (3) reduced venous blood supply. These responses are aimed at sustaining arterial and venous pressure as well as cardiac output (formula 1) (Table 1) [52].

**Table 1:** Practical information about the quantitative bleeding range in different laboratory species

Animal species	Average weight (g)	Blood volume (ml/kg)	Adult total blood volume (ml)	Volume of safe blood sampling in a blood vessel (ml)	Extractable whole blood (ml)
Syrian mice	18–40	58.5	1.7	0.1–0.2	0.8–1.4
Hamster	85–150	78	7.8	0.5	0.2–1.5
Rat	250–500	54–70	23	2.25	10.7
Rabbit	1000–6000	57–65	58.5–585	5–50	31–310
Dog	-	70–110	900–1170	90–110	-
Sheep	-	58–65	4060–4480	400–450	-
Pig	-	56–69	4200–4800	420–480	-

$$\:total\:blood\:volume=\frac{plasma\:volume\:\times\:100}{100-(0.96\times\:hematocrite)}$$

Formula 1: Total blood volume estimation, 0.96 is the coefficient of plasma trapped among red blood cells.

Hemorrhage is classified as moderate when 15 to 20% of the total blood volume is lost. Consequently, it is not feasible to maintain arterial and venous pressure, nor cardiac output, which will decline. Under these new conditions, fluids enter the bloodstream from the skin surface and gastrointestinal tract. Additionally, insufficient oxygen exacerbates anaerobic glycolysis, leading to acidosis and elevated respiration rates [52].

In cases where injury results in the loss of 30 to 40% of total blood volume, an acute phase of shock may occur. This phase is marked by coldness of the skin and extremities, weak and rapid pulse, dehydration, restlessness, muscle weakness, increased respiratory rate, and decreased body temperature. This process resulted in the disruption of the tissue’s blood supply and extensive cell death, ultimately leading to death [52].

While the aforementioned classification encompasses the general processes of the body’s response to hemorrhage, notable variations have been observed across different species. Hemorrhage-related mortality in species such as pigs occurs less frequently compared to other animals. Animal responses to 20% hemorrhage under pentobarbital anesthesia are comparable to those observed at 40% hemorrhage regarding levels of consciousness. The following table illustrates the responses of various laboratory species to different percentages of hemorrhage (Table 2) [52].

**Table 2:** Reaction of some laboratory animal strains to different percentages of hemorrhage

species	rat									
Fractional total volume loss (percentage)	5–10	11–20			20–30			Near 40		More than 50
Clinical signs and laboratory changes	Possible changes in Plasma corticosterone and prolactin concentrations	50% reduction in cardiac output			25% reduction in core arterial pressure			Gastrointestinal necrosis		85% reduction in cardiac output, usually without recurrence
reference	[64]	[65]			[65]			[66]		[67]
species	dog									
Fractional total volume loss (percentage)	30 >			30–40					> 40	
Clinical signs and laboratory changes	Maintain blood pressure for 24 h and normal blood volume for up to 90 min			20–23 mmHg reduction in blood pressure, Increased heart rate, Decreased cardiac output					Two-thirds reduction in arterial pressure, death within 2.5 h	
reference	[68]			[52]					[51]	
species	pig									
Fractional total volume loss (percentage)	Near 10		In the range between 20 to 30						Near 40	
Clinical signs and laboratory changes	Mortality rate is 17%		Mortality rate is 66%						Mortality rate is 88%	
reference	[69]									
species	human									
Fractional total volume loss (percentage)	Range 17					30–40				
Clinical signs and laboratory changes	Clear reduction in all cardiac indices including blood volume, main arterial pressure and central venous pressure						Shock due to hemorrhage			
reference	[70]						[71]			

### Blood and tissue indices evaluation

#### Pre-test assessments

Animals, particularly those that are genetically non-inbred, must be evaluated and confirmed for the normalization of coagulation-related tests, including prothrombin time (PT), thromboplastin activation time (PTT), platelet count, clotting time, and fibrinogen activity. Additionally, these variables may be re-evaluated post-experiment for statistical analysis [37].

#### Confirmation of hemorrhage and grading through observation

The assessment of hemorrhage is contingent upon the species of the animal, the location of the injury, and the rate of blood outflow. Observation and confirmation of hemorrhage using visual assessment for a duration of 5 to 45 s is effective for determining injury and documenting the potential efficacy of hemostatic agents in subsequent stages. The efficacy of each hemostatic agent is evaluated by monitoring for hemorrhage at 15 to 30-second intervals following its application. Based on contractual principles, if observation persists for 90 to 120 s without cessation of bleeding, the materials under study are not classified as a hemostat. In cases involving major arteries, the final time determination for complete cessation of flow may be extended and monitored for up to ten minutes [53].

**Fig. 3:**
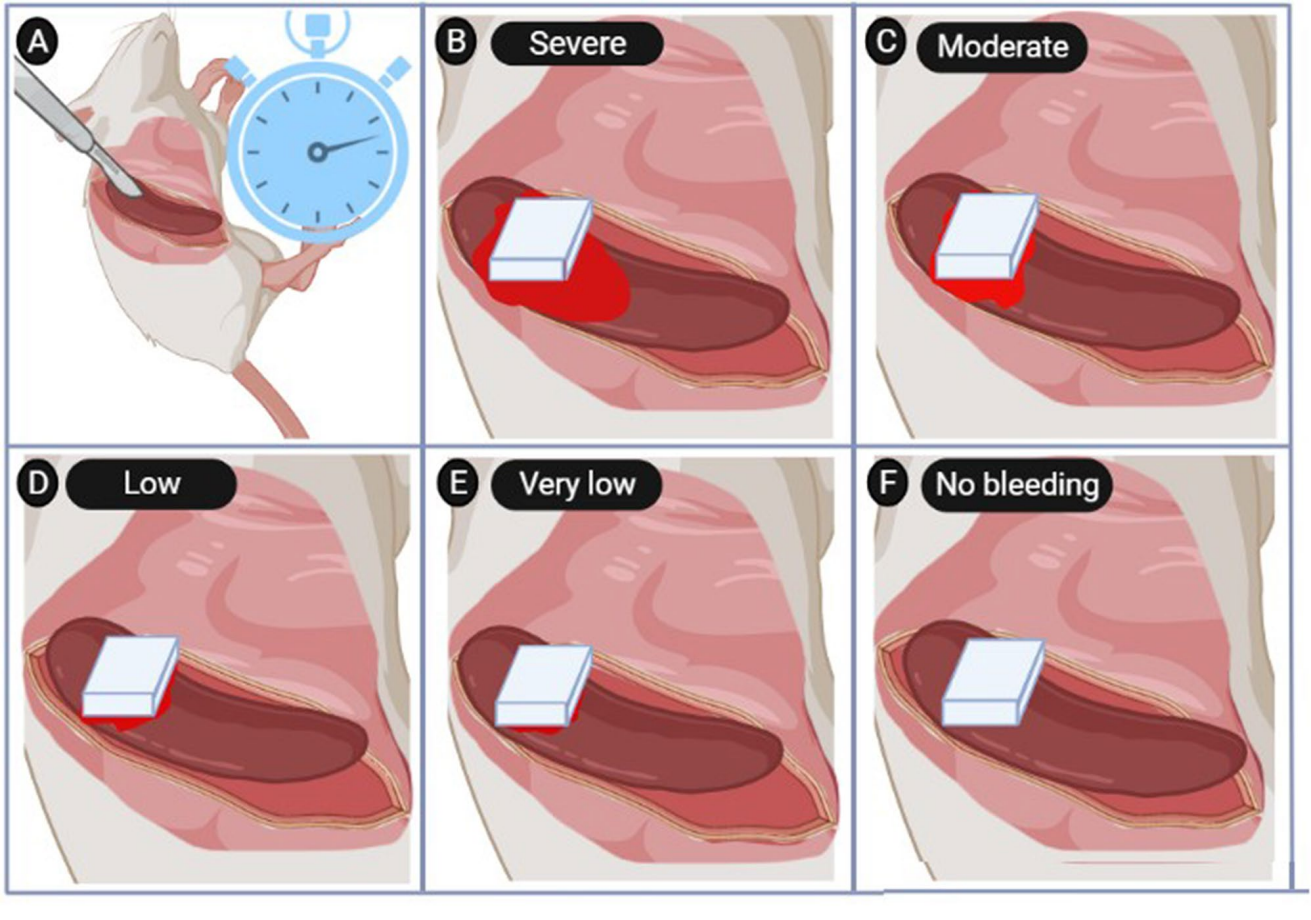
Visually hemorrhage quality-rating system in 5 step by considering time

The initial step in hemorrhage grading, aside from the primary arteries of the body like visceral tissues, relies on ocular observations as outlined below: Zero degrees indicates no visible blood; first degree shows dripping blood at the injury margin; second degree presents slow blood flow; third degree indicates definite hemorrhage; fourth degree reveals continuous and visible hemorrhage at the injury site (Fig. 3) [54].

**Fig. 4:**
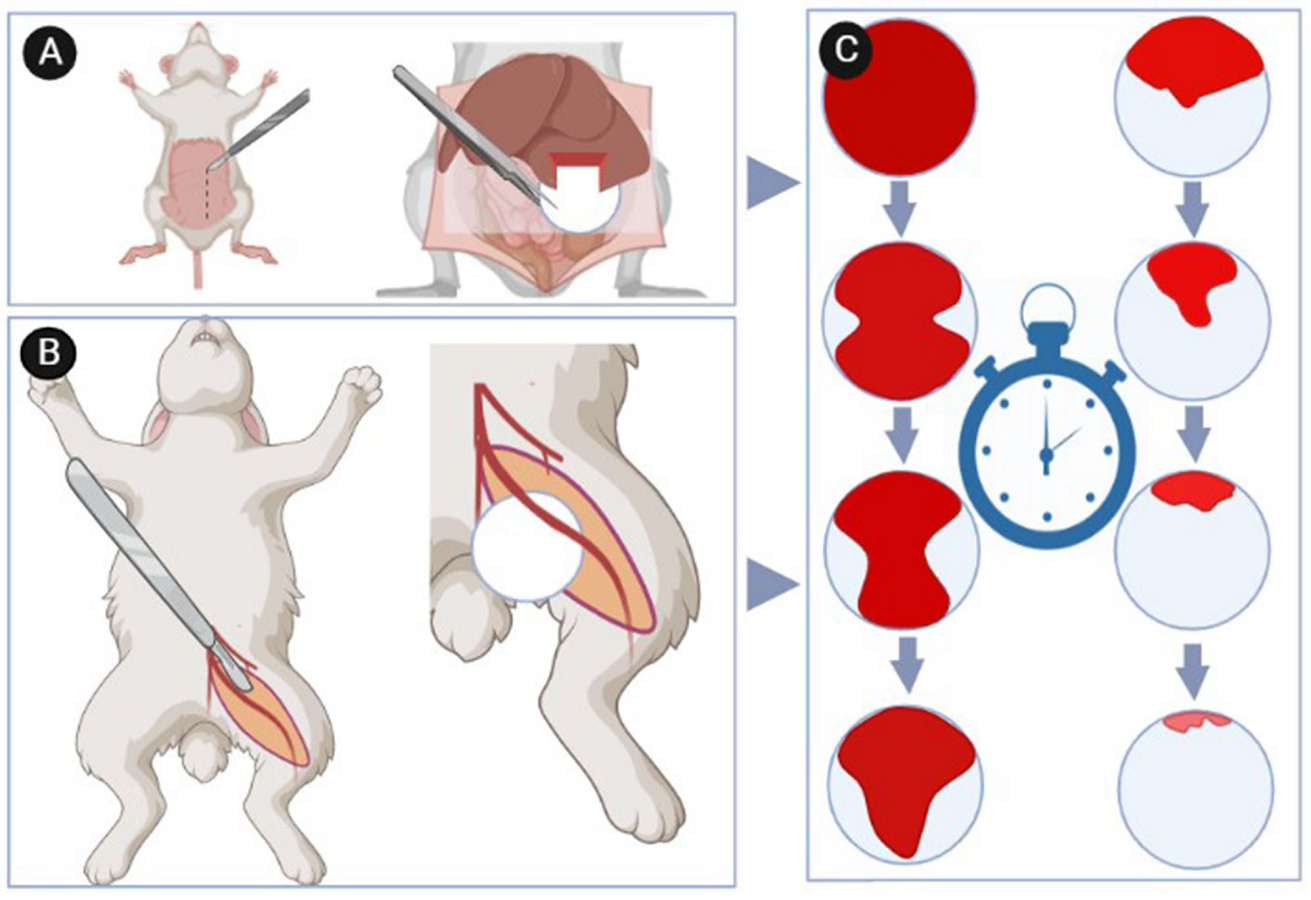
Hepatic hemorrhage modeling by razor blade or scalpel in rat and non-compressible hemostat test (**A**); femoral vein hemorrhage modeling in rabbit and compressible hemostat test (**B**); evaluation the bleeding volume by using similar size and shape filter paper within time (**C**)

Research indicates that the estimation of hemorrhage volume and speed, along with group comparisons, is more precise when utilizing filter paper. In this procedure, after blood collection, circular plates of uniform size may be cut from filter paper and placed at the injury site. Once blood has fully diffused into the control group paper, all paper groups are altered, and this procedure is maintained at consistent time intervals until hemorrhage ceases and no additional blood is released in the experimental groups. Hemorrhage duration can be calculated by counting the number of papers and multiplying by specified intervals. Additionally, the lost blood volume is determined by subtracting the initial weight of the consumed paper in each group from the total weight of the blooded paper. This method is more effective than the observational strategy, particularly for main artery hemorrhage. Additionally, it is relevant for tissue hemorrhage (Fig. 4) [53].

### Main and common variables in a hemorrhage model

The primary variable in any experimental hemorrhage study is the volume of blood loss. The variable is primarily determined by directly weighing the blood collected from the injured site using a suction system. In instances involving humidity absorbent materials, the trapped blood content is calculated by subtracting the post-experimental hemostat weight from the pre-experimental weight. Moreover, the estimation of hemorrhage volume can be correlated with the blood loss observed in experimental groups, provided that the hemostatic agent is maintained at the hemorrhage site. The results contribute to the assessment of lost blood volume and rate across each group of repetitions, as well as within underrepresented groups [14].

Main arterial pressure (MAP) and the varying heart rate, both consistently measured during the test using blood pressure monitors, represent significant variables. Researchers have examined blood microscopic variables, including thromboelastographic and coagulation-dependent factors, the total and dissociation number of cells in the bloodstream, as well as the concentration and pressure of gases, particularly oxygen and carbon dioxide.

### Side variable in hemorrhage modeling

In addition to the previously mentioned blood markers, other variables such as the quantity and morphology of red and white blood cells, along with various biochemical indices including blood hemoglobin (Hb), hematocrit (Hct), glucose, and electrolytes, can also be assessed.

In organ hemostatic studies, the estimation of blood flow for each organ is commonly assessed by measuring the inflow and outflow of blood from the primary vessels [35]. Additionally, circulating blood products from organs intended for hemorrhage modeling and hemostasis warrant investigation in research studies.

### Hemostat agent related indices

Hemostatic agents require evaluation of various characteristics, including their interaction with moist wound environments and the overall body environment [55]. The primary assessments that facilitate this objective are including:1) After exposure to non-ionized water, the hemostatic agent’s weight is subtracted from its raw weight in a swelling ratio (water absorption) examination, 2) Tiny amounts of the test agent are placed in wells of commercial standard cell culture to measure cell death, 3) Red blood cells and platelet particles binding capability to the hemostatic agent by mixing with heparinized blood and platelet-rich plasma (PRP), then washing, weighing, and confirming with the help of electron microscopy (SEM), 4) Measurement of hemostatic agent disintegration in vitro and in vivo by subtracting the initial dry weight from the final dry weight in cell culture medium combined physiological coordinates medium and implantation in the peritoneal area of mice or rats at intervals of several days, 5) Potential allergic reactions assessment was done by subcutaneous injection of test materials in mice and observation of macroscopic and microscopic reactions, and 6) Evaluation of the hemostatic agent antimicrobial capability was performed by placing its plates in the culture medium with frequent wound contaminants such as Staphylococcus aureus or treatment of *S. aureus* infected wounds with hemostatic agents.

If we plan to explore the interactions between a substance and body tissue after a long period, wound tissue sampling and subsequent routine and specific staining for investigating inflammation and angiogenesis are required following experimental study completion. These checks include: (1) In a swelling ratio examination, the weight of the hemostatic agent is subtracted from its raw weight following exposure to non-ionized water to assess water absorption, (2) Small quantities of the test agent are introduced into wells of commercially standardized cell culture to assess cell mortality, (3) The binding capability of red blood cells and platelet particles to the hemostatic agent was assessed by mixing with heparinized blood and platelet-rich plasma (PRP), followed by washing, weighing, and confirmation through electron microscopy (SEM), (4) Measurement of hemostatic agent disintegration in vitro and in vivo involves subtracting the initial dry weight from the final dry weight in cell culture medium, combined with physiological coordinate’s medium, and implantation in the peritoneal area of mice or rats at intervals of several days, (5) Assessment of potential allergic reactions is conducted through subcutaneous injection of test materials in mice, followed by observation of both macroscopic and microscopic reactions, and (6) The antimicrobial capability of the hemostatic agent was evaluated by placing its plates in culture medium contaminated with frequent wound pathogens, such as Staphylococcus aureus, or by treating *Staphylococcus aureus* infected wounds with hemostatic agents.

To investigate the interactions between a substance and body tissue over an extended period, it is necessary to conduct wound tissue sampling and perform routine and specific staining to assess inflammation and angiogenesis after the completion of the experimental study.

### Hemostat agents

Clotting is defined as a natural post-injury process in which blood pressure drops and clots form to prevent further bleeding. Normal clotting includes vascular contraction, clot formation, and fibrin adhesive network formation for emerging clots at the outlet of the vessel [56, 57]. It has been illustrated that implementing a functional hemostatic agent as soon as possible is the first goal in severe hemorrhagic injury. Therefore, any effort should be made to speed up the preceding steps [58].

An effective hemostatic agent at the accident site should meet the following criteria: (1) Rapid and effective control of hemorrhage upon application to the wound; (2) Acceptable outcomes even in the presence of active hemorrhage (pool formation); (3) Should be administered in the shortest possible time without the need for mixing; (4) Usable by patients with minimal training in medical emergencies. (5) Compact in size and weight; (6) Capable of being stored at room temperature for a minimum of two years and usable for several weeks under working conditions, specifically within a temperature range of -10 to + 55 degrees Celsius; (7) Must be entirely free of adverse effects, ensuring safety and not causing any wounds; (8) Prevent penetration and inhibit microorganism growth to the greatest extent possible; (9) Should be cost-effective; and (10) Maintain hemostatic properties for a minimum duration of several hours until the injured individual reaches a medical facility [59, 60].

In addition to compressing the wound area or serving as a bandage, cellulose-based cloth gauze and commercial and military hemostatic agents, such as HemCon and QuickClot, are commonly employed in the management of hemorrhagic lesions. Additionally, they may serve as control groups for researchers’ innovative hemostatic agents [61].

Recent investigations reveal that the selection of hemorrhage model in experimental studies and the choice of hemostatic agent are influenced by the physical properties of the hemostat. Solid and flat hemostatic agents or cellulosic fabric additives are primarily utilized for compressible hemorrhages [62]. Round, liquid, and paste hemostats are primarily utilized for managing hemorrhage in areas that are difficult to access. Moreover, high-accuracy and energy-dependent hemostatic agents such as plasma and ultrasound are limited to surgical operating rooms [24, 63]. Applying direct pressure to the hemorrhage area is the initial step in managing cutaneous hemorrhage (compressible). Consequently, placing weight on the hemostatic agent is a common practice in cutaneous hemorrhage modeling. The recommended weight range is 50 g for rats, several hundred grams for rabbits, and a few kilograms for farm animals.

Despite the continued use of sutures for main vein closure and gassing in wound sites in numerous surgical settings, their limitations have prompted the development of a novel hemostatic instrument. This instrument offers enhanced accuracy, increased hemostatic reliability, and improvements in surgical speed and quality. The performance of these devices varies significantly, with each research group utilizing a distinct array of features, including electric blades, carbon dioxide lasers, ultrasound, electromagnetic waves, and plasma, particularly in one of the less-explored hemorrhagic models [41].

## Conclusions

Recent investigations reveal that hemorrhage accounts for over 40% of civilian trauma deaths and more than 90% of preventable military deaths. Additionally, significant hemorrhage-related fatalities from traumatic incidents, such as accidents, transpired within the initial two and a half hours. The statistics indicate that the trauma-time ratio is a significant issue; however, they do not comprehensively address all facets of the problem. Hemorrhage and the need to counteract its effects during surgery present significant challenges that require further research in hemorrhage modeling and hemostatic agents. This study demonstrates that models exhibiting greater resemblance to real settings yield more substantial conclusions about various types of homeostatic materials and equipment. Moreover, integrating physical and biochemical characteristics in experimental research design might enhance the reliability and accuracy of homeostatic processes.

## Data Availability

All data and material are available in article.

## Abbreviations

Hct: Hematocrit
Hb: Blood hemoglobin
MAP: Main arterial pressure
PRP: Platelet-rich plasma
PT: Prothrombin time
PTT: Thromboplasmin activation time
SEM: Electron microscopy
TBV: Total blood volume
